# Exploring the lead-free halide Cs_2_MGaBr_6_ (M = Li, Na) double perovskites for sustainable energy applications

**DOI:** 10.1038/s41598-024-54386-1

**Published:** 2024-03-06

**Authors:** Mudasir Younis Sofi, Mohd Shahid Khan, Javid Ali, M. Ajmal Khan

**Affiliations:** Department of Physics, Jamia Millia Islamia, New Delhi, 110025 India

**Keywords:** Structural and thermodynamic stability, Electronic properties, Thermoelectric coefficients, Electron–phonon coupling, Optical parameters, Computational methods, Electronic structure, Thermoelectrics, Physics

## Abstract

In recent years, there has been a growing emphasis on the exploration of sustainable and eco-friendly materials well-suited for advanced applications in the realms of thermoelectrics and optoelectronics. Lead-free halide double perovskites have emerged as a compelling class of materials in this context. Nevertheless, despite their potential utility, thorough investigations into their thermal transport characteristics remain limited. In this systematic investigation, we employ density functional theory (DFT) and post-DFT techniques to elucidate the essential stability parameters, transport properties, and carrier-lattice interactions of the metal halide-based Cs_2_MGaBr_6_ (X = Li, Ga) double perovskites. Our assessment of structural stability involves a meticulous description of stability index parameters and the optimization of pristine structures using the GGA-PBE potential. Additionally, we calibrate the electronic structure while taking spin–orbit coupling (SOC) effects into consideration by using a combination of GGA and GGA + mBJ potentials. Our findings reveal that the TB-mBJ derived band gaps of 1.82 eV and 1.78 eV for Cs_2_LiGaBr_6_ and Cs_2_NaGaBr_6_ reside within the visible spectrum, prompting further investigation into their thermal transport characteristics. Moreover, we analyze the phonon characteristics and vibrational modes, extending our investigation to examine the electron–phonon coupling strength. The scrutiny of the Fröhlich coupling constant and the Feynman polaron radius unveils a stronger electron–phonon coupling strength. In the domain of thermoelectrics, the significant figure of merit (zT) values of 1.08 and 1.04 for Cs_2_LiGaBr_6_ and Cs_2_NaGaBr_6_, respectively, emphasize the considerable potential of these materials for deployment in renewable energy applications. Furthermore, our computational investigation into optical properties, including the dielectric constant, optical absorption, and refractive index, demonstrates optimal performance within the visible spectrum. Specifically, elevated absorption coefficient values of $30\times 10$^4^ ${{\text{cm}}}^{-1}$ for Cs_2_LiGaBr_6_ and $40\times 10$ ^4^ ${{\text{cm}}}^{-1}$ for Cs_2_NaGaBr_6_ are noted across visible and infrared spectra, highlighting their promising potential in optoelectronic and solar cell technologies.

## Introduction

The rising global energy requirements have resulted in a significant upsurge in the utilization of fossil fuels, a primary contributor to environmental pollution and global warming. The pressing need for sustainable energy sources to address this crisis has propelled silicon-based solar cells into the spotlight as a potential solution. Nevertheless, the intricate manufacturing processes and constrained efficiency of these solar cells have redirected research efforts toward the exploration of sustainable and economically viable alternative photovoltaic (PV) materials. These photovoltaic materials (PV) facilitate the direct conversion of sunlight into electricity through the utilization of semiconductor materials in solar cells. Over the past few decades, perovskite solar cells have shown remarkable progress, elevating their efficiency from a mere 3.8% in 2009 to an impressive 22.7% by 2017, albeit in laboratory settings^1–4^. Recent progress in the field of halide perovskites, particularly inorganic-lead halide perovskites exemplified by CH_3_NH_3_PbI_3_, has attracted considerable interest owing to their remarkable optical and photovoltaic characteristics^1,5^. Nevertheless, the practical utilization of these materials is impeded by challenges such as lead toxicity and enduring stability concerns, necessitating exploration for lead-free substitutes. These alternatives must be cost-effective, easily recyclable, and possess outstanding optoelectronic properties. Additionally, they must meet commercial requirements such as flexibility, long-term stability, and scalability, while also competing with established PV technologies^6–8^. To address these challenges, a significant focus has been placed on substituting lead-based perovskites with environmentally friendly lead-free halide-based perovskites. The success of this approach is attributed to the remarkable optoelectronic properties exhibited by these semiconducting halide perovskites, including a high optical absorption coefficient, tuneable bandgap, long carrier recombination lifetimes, high carrier mobility, small electron/hole effective masses, and a high molar extinction coefficient. Recent theoretical and experimental studies have focused on investigating the stability and potential optoelectronic applications of these lead-free alternatives^9–11^. In this context, numerous compounds have been explored via a first principles approach, including alkali metal combinations (Li, K, Na, Cs), specific cations such as In and Ag, and elements like Bi and Sb in the M^3+^ state, as well as halogens like Cl and Br, with a focus on their diverse applications^12–15^. However, recent attention has shifted towards combinations of Li, Na, and K with Ga, In, and Tl-based compounds, recognized for their inherent stability, direct band gap properties, and favourable electron/hole carrier mobility characteristics. A notable study by M. Zia et al.^16^ employed ab initio simulations to investigate the thermal and dynamical stability of metal halide-based Cs_2_LiTlBr_6_ and Cs_2_NaTlBr_6_ compounds. The findings revealed the decisive thermal and dynamic stability, along with a direct band gap characteristics and small electron/hole carrier mass, positioning them as promising candidates for applications in transport dynamics and optoelectronics. Furthermore, significant research efforts have centered around first principles calculations of metal halide-based compounds such as Cs_2_XInI_6_ (X = Li, Na)^17^, Cs_2_B'BiI_6_ (Li, Na)^18^, and Cs_2_InGaX_6_ (Cl, Br)^19^ halide perovskites. These studies emphasize the structural stability, resistance against decomposition, and ideal band gap values of these materials, contributing to their exceptional optical performance within the visible spectrum.

In addition to photovoltaic (PV) technology, recent decades have witnessed significant interest in thermoelectric advancements due to their capacity to efficiently harness waste heat for electricity generation, thus addressing energy efficiency concerns and fostering sustainable power generation. The thermoelectric efficiency of a material is quantified through the dimensionless figure of merit (zT), expressed as: ${\text{zT}}=\frac{{S}^{2}\sigma }{{\upkappa }_{{\text{e}}}+{\upkappa }_{{\text{l}}} }{\text{T}}$, where S denotes the Seebeck coefficient, σ represents electrical conductivity, *T* signifies absolute temperature, κ_e_ implies electronic thermal conductivity, and κ_l_ refers to lattice thermal conductivity^20,21^. A material is considered suitable for thermoelectric applications when it exhibits a zT value approximately equal to 1.0, achieved through a combination of a high-power factor (PF) and low thermal conductivity^22^. Surprisingly, despite their low thermal conductivity due to cation arrangement and high charge mobility, halide double perovskites have mainly been studied for optoelectronic applications, with limited experimental focus on their thermoelectric efficiency. Nevertheless, there is increasing interest in exploring the thermoelectric properties of halide perovskites. Theoretical calculations have indicated that halide hybrid perovskites can potentially achieve a ZT value of one, and indeed, several perovskite compounds, including MASnI_3_^23^ (ZT ~ 1.0), Cs_2_InAgX_6_^24^ (ZT ~ 1.0), and Cs_2_KTlX_6_^25^ (ZT ~ 0.85), have reached a figure of merit equal to one. In a recent study conducted by Tariq M. Al-Daraghmeh et al.^17^, an investigation on metal halide-based Cs_2_XInI_6_ (X = Li, Na) double perovskites revealed a ZT value of 1.00 for each material at room temperature, indicating their potential for practical energy harvesting devices.

In light of the above literature, we performed first-principles calculations to explore the relevant stability, transport properties, and polaronic properties of metal halide-based Cs_2_MGaBr_6_ (M = Li, Na) double perovskites. These materials have been identified as promising candidates for optoelectronic applications by Zia et al.^26^. However, there has been a lack of inclusive research into the structural, thermodynamic, and dynamic stability of these materials, and no prior studies have explored their thermoelectric properties. Consequently, we performed simulations employing density functional theory (DFT) and post-DFT methods to comprehensively investigate the stability, transport characteristics, and carrier-lattice interactions of Cs_2_MGaBr_6_ double halide perovskites.

## Results and discussions

The anticipated stability parameters, electronic, thermoelectric and carrier-lattice interactions of Cs_2_MGaBr_6_ halide double perovskites are discussed below.

### Structural and thermodynamic stability

**Figure 1:**
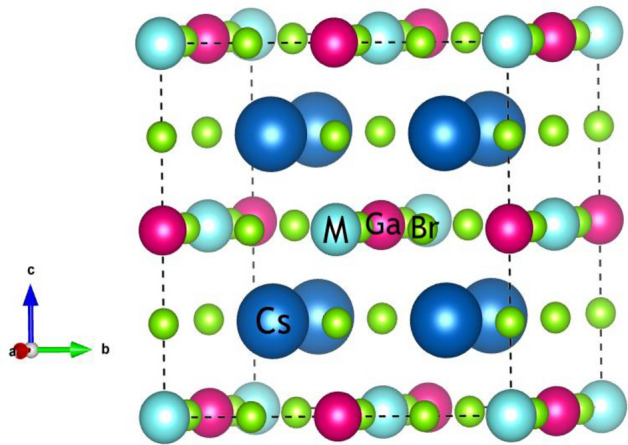
Crystal structure of Cs_2_MGaBr_6_(M = Li, Na) halide perovskites.

The structural configuration of Cs_2_MGaBr_6_ (M = Li, Na) conforms to the Fm-3m cubic stability. In this arrangement, cesium (Cs) is enclosed within a cage formed by twelve halide atoms (Br), while sodium (Na) and gallium (Ga) atoms are situated in an octahedral coordination with halide atoms. Each of these atoms exhibit a coordination number of six with the surrounding halide atoms. A visual representation of this crystal structure and the spatial occupation of atoms is illustrated in Fig. 1.

Firstly, we commence by evaluating the structural stability of the given materials through the utilization of diverse stability index parameters. Double perovskites characterized by the general stoichiometry A_2_BB'X_6_ typically adhere to an ideal cubic crystal structure. However, deviations from this idealized structure may arise owing to factors such as size effects, octahedral tilting, and Jahn–Teller distortions. In order to uphold cubic stability in Cs_2_MGaBr_6_ halide perovskites and mitigate structural deformations, we employed two crucial stability assessment parameters, specifically the Goldschmidt tolerance factor and the octahedral factor. The tolerance factor, accounting for size effects in A_2_BB'X_6_ halide crystals, and the octahedral factor, assessing overall stability, are expressed as $: {t}_{dp}=\frac{{r}_{A}+{r}_{X}}{\sqrt{2}{r}_{BB}{\prime}+{r}_{X}}$**,** µ**=** ${r}_{B}/{r}_{X}$ where ${r}_{A}$, ${r}_{B}$, and ${r}_{X}$ are the Shannon ionic radii for A^+^, B^+^, B' and X^−^ ions, respectively^27,28^. For stable cubic perovskites, the ranges of ${t}_{dp}$ and µ are 0.8 ≤ ${t}_{dp}$  ≤ 1.0 and 0.29 ≤ µ ≤ 0.55. The calculated values in Table 1 show that the considered perovskites are stable in cubic structures at room temperatures.

**Table 1:** Intended values of tolerance factor (τ) (unitless), octahedral factor (µ) (unitless), optimsed lattice parameter (a_0_ in Å), bulk modulus (B in GPa), pressure derivative of bulk modulus (B_0_') (unitless), unit cell volume (V_0_ in a.u^3^), and ground state energy (E_0_ in eV) for Cs_2_MGaBr_6_ halide perovskites.

Configuration	τ	µ	a_0_	Other works		B	B_0_'	V_0_	E_0_
				Theory	Exp				
Cs_2_LiGaBr_6_	1.00	0.36	10.75	10.78^26^, 10.96^16^	11.42 ^30^	23.23	4.34	2094.04	− 902,294.33
Cs_2_NaGaBr_6_	0.98	0.42	10.95	10.96^26^, 11.13^16^	11.42 ^30^	22.09	4.36	2218.93	− 906,507.43

**Figure 2:**
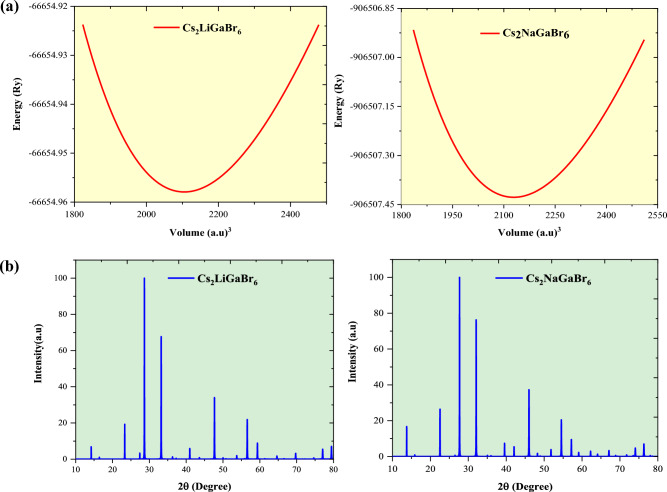
(**a**) Graphical depiction of the optimized volume-energy relationship, and (**b**) DFT-simulated XRD patterns of Cs_2_MGaBr_6_ (M = Li, Na) double halide perovskites.

Subsequent to the stability assessment, the ensuing step entails the determination of key structural parameters, encompassing the equilibrium lattice constant (a_0_), the bulk modulus (B), and its corresponding pressure derivative (B'_0_). This is accomplished through the computation of the total energy as a function of the unit cell volume (*E*(*V*)). To scrutinize this correlation, a fitting procedure is applied utilizing the Birch-Murnaghan equation, formulated as a second order expression, given as^29^: $E(V)={E}_{0}+\frac{9{B}_{0}{V}_{0}}{16}\left\{\left[{\left(\frac{{V}_{0}}{V}\right)}^{2/3}-1\right]\right.B{^{\prime}}_{0}+{\left[{\left(\frac{{V}_{0}}{V}\right)}^{2/3}-1\right]}^{2}\left.\left[6-4{\left(\frac{{V}_{0}}{V}\right)}^{2/3}\right]\right\}$. In this context, the symbol $E(V)$ represents the ground state energy corresponding to the cell volume *V*, with *V*_0_ representing the equilibrium volume. The term *E*_0_ signifies the total equilibrium energy, while B and B'_0_ denote the bulk modulus and its pressure derivative, respectively. The optimized volume-energy plots, as illustrated in Fig. 2a, facilitate the determination of crucial structural parameters, including the lattice constant (a_0_), bulk modulus (B), and its pressure derivative (B'_0_), achieved by identifying the minimum point on the *E*(*V*) curve. The computed values of all these parameters are presented in Table 1, along with previously published experimental and theoretical results for analogous materials. Significantly, the generalized gradient approximation (GGA) approximation yields a lattice parameter value that closely aligns with the experimental value and other theoretical findings^16,26,30^. Furthermore, X-ray diffraction (XRD) spectra for Cs_2_MGaBr_6_ perovskites, utilizing the optimized structures, have been generated through simulations conducted with the VESTA Crystallographic Software, as depicted in Fig. 2b. The simulated X-ray diffraction (XRD) patterns exhibit notable concordance with experimentally obtained XRD patterns of analogous materials, like Cs_2_NaBiBr_6_ halide perovskite^30^, providing valuable insights for advancing further experimental investigations into these materials.

In addition to the structural stability, the thermodynamic stability of the specified materials has been evaluated by computing the enthalpy of formation per atom at absolute zero temperature, expressed by the equation^21^: ΔH = ${E}_{{Cs}_{2}MGa{Br}_{6}}-$ ($2{E}_{Cs} +{E}_{M}+ {E}_{Ga}+ 6{E}_{Br}$). Here, ${E}_{{Cs}_{2}MGa{Br}_{6}}$ represents the equilibrium energy of the Cs_2_MGaBr_6_ (M = Li, Na) materials, and ${E}_{Cs}$, ${E}_{M}$ ${E}_{Ga}$, and ${E}_{Br}$ are the total energies of cesium, lithium/sodium, gallium, and bromine atoms in their stable elemental crystal structures. The computed values of ΔH are − 1.56 eV and − -1.67 eV for Cs_2_LiGaBr_6_ and Cs_2_NaGaBr_6_, respectively. The negative values of the computed enthalpy of formation indicate the thermodynamic feasibility of experimental production and synthesis for the given materials.

### Mechanical stability

In the pursuit of practical applications, effective elastic constants play a pivotal role in determining the structural stability and the material's response to external forces. Employing the Cubic Elastic package developed by Thomas Charpin^31^, we projected the second-order elastic constants (SOECs) and subsequently evaluated the mechanical properties of the investigated double halide perovskites. The equilibrium cubic structure undergoes deformation through the application of small strains, allowing us to predict second-order elastic constants. The mechanical stability of the cubic structure relies on the deformed structures possessing higher energy levels than the original cubic phase, leading to specific limiting conditions for elastic constants^32^: ${C}_{11}-\left|{C}_{12}\right| > 0$; ${C}_{12} < B < {C}_{11};$ ${C}_{44} > 0;$ ${C}_{11}>0; {C}_{11}+2{C}_{12}>0.$ The obtained elastic constants, specifically C_11_ (representing longitudinal elasticity along the unit cell axis), C_12_, and C_44_ (shear elastic constants governing shape elasticity), play a pivotal role in anticipating the material's response to applied stresses. Herein, the second-order elastic constants (SOECs) have been systematically evaluated employing the energy-strain approach within the framework of GGA-PBE, as detailed in Table 2. Remarkably, all three elastic constants are non-negative and satisfy the Born stability criteria, confirming the mechanical stability of the given materials. Utilizing the second-order elastic constants (SOEC) within the Voigt-Ruess Hill scheme^33–35^, we have computed several elastic parameters, including Young's modulus (Y), shear modulus (G), bulk modulus (B), and Poisson's ratio (ν), employing mathematical expressions documented elsewhere^21^. Notably, the C_11_-values for both compounds surpass those of the other two shear elastic constants, C_12_ and C_44_. Likewise, the fact that that the bulk modulus value (B) exceeds the shear modulus value (G) signifies heightened resistance to volumetric deformation compared to shape deformation in these materials. Moreover, employing the second-order elastic constants (SOECs), anisotropic parameters including Zener (A_Z_) and Universal anisotropic parameters (A_U_) have been determined using the equations as follows^36,37^: ${{\text{A}}}_{{\text{Z}}}=1+\frac{(2{C}_{44}+{C}_{12})}{{C}_{11}}$, ${{\text{A}}}_{{\text{U}}}=5\frac{{G}_{V}}{{G}_{R}}-5$]. The calculated values of these parameters are listed in Table 2. The Zener elastic parameter, primarily linked to shear anisotropy in materials, remains fixed at 1 in isotropic materials. However, any deviation from unity, as seen in both A_U_ and A_Z_ in Table 2, signifies the presence of anisotropic behaviour in the studied materials. The Kleiman parameter (ξ) has been subsequently determined from the elastic constants utilizing the equation^21^: $\upxi =\frac{{C}_{11}+8{C}_{12} }{7{C}_{11}+2{C}_{12}}.$ The observed low value of ξ signifies an enhanced resistance of these materials to bond bending and bond angle distortions (Table 2).

**Table 2:** Calculated second order elastic constants ${{\text{C}}}_{11}$, ${{\text{C}}}_{12}$, and ${{\text{C}}}_{44}$ (GPa);) bulk modulus B (GPa); shear modulus G(GPa) Young’s modulus Y (GPa); Poisson’s ratio ν (unitless); B/G ratio; Cauchy’s pressure (${{\text{C}}}_{12}-{{\text{C}}}_{44}$) (unitless), Kleiman’s parameter ($\xi )$ (unitless), Zener anisotropic constant (A_z_) and universal anisotropic constant(A_U_) of Cs_2_MGaBr_6_ double halide perovskites.

Parameter	*C*_*11*_	*C*_*12*_	*C*_*44*_	*B*	*G*	Y	*v*	*B/G*	*C*_*12*_*–C*_*44*_	*ξ*	*A*_*z*_	*A*_*U*_
Cs_2_LiGaBr_6_	45.52	18.95	18.98	27.56	16.32	40.08	0.25	1.68	− 0.03	0.55	1.42	0.14
Cs_2_NaGaBr_6_	39.75	14.18	14.56	22.70	13.56	32.81	0.25	1.67	− 0.38	0.42	1.13	0.02

To evaluate the stability of these materials at elevated temperatures, we ascertained their melting temperature utilizing the equation^21^: ${T}_{m}(K)=\left[553(K)+\left(5.911\right){C}_{11}\right]GPa\pm 300$. The predicted melting temperatures are determined to be 822.06 ± 300 K for Cs_2_LiGaBr_6_ and 787.96 ± 300 K for Cs_2_NaGaBr_6_, respectively. Notably, the high value of the melting temperature suggests that these materials exhibit stability even under extreme temperatures.

To gain a deeper understanding of the mechanical stability of these perovskite materials, it is imperative to evaluate their ductility or brittleness characteristics, often dynamically predicted through parameters such as the Cauchy pressure factor, Pugh ductility index, and Poisson's ratios^21,38,39^. In Table 2, the computed parameters, including Pugh's ratio (B/G), Poisson's ratio (ν), and Cauchy pressure (C_12_–C_44_), consistently demonstrate values below critical thresholds (1.75, 0.26, and 0,) respectively, signifying the inherent brittleness of the studied materials. Additionally, the determination of longitudinal and transverse elastic wave velocities has been achieved by utilizing pertinent bulk and shear values, in conjunction with the density of these compounds, as outlined in scientific formulations^20^: ${v}_{t}={\left[\frac{G}{\rho }\right]}^\frac{1}{2} and {v}_{l}={\left[\frac{3B+4G}{3\rho }\right]}^\frac{1}{2}$. The calculated longitudinal (transverse) wave velocities for Cs_2_LiGaBr_6_ and Cs_2_NaGaBr_6_ in (*ms*^*-1*^) are 1926 (3351.11) and 1791.25 (3111.26), respectively.

**Figure 3:**
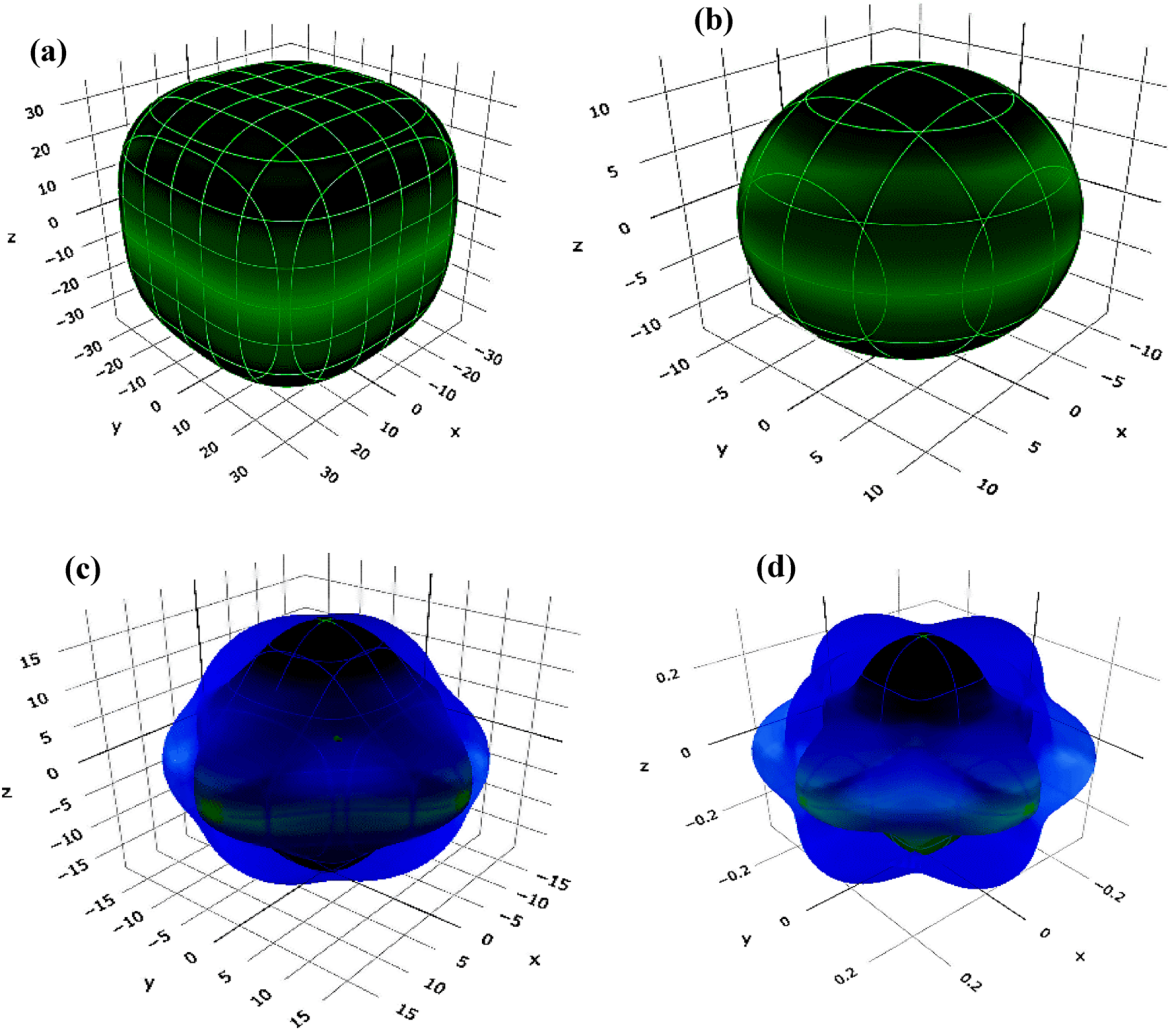
Three-dimensional representation of (**a**) Young's modulus (*Y*), (**b**) linear compressibility (β), (**c**) shear modulus(*G*), and (**d**) Poisson's ratio (*v*) for Cs_2_LiGaBr_6_ halide perovskite.

**Figure 4:**
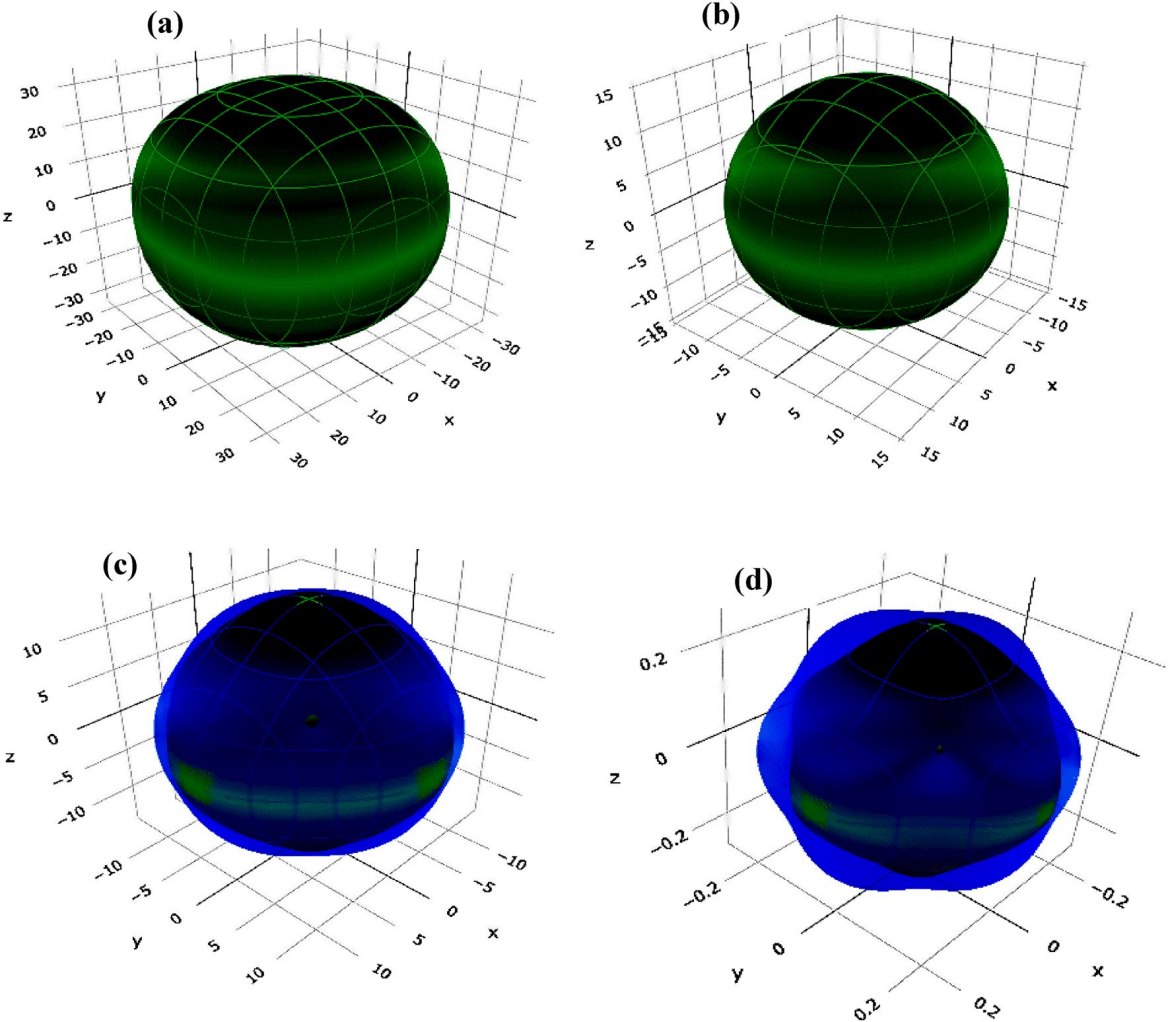
Three-dimensional representation of (**a**) Young's modulus (*Y*), (**b**) linear compressibility (β), (**c**) shear modulus (*G*), and (**d**) Poisson's ratio (*v*) for Cs_2_NaGaBr_6_ halide perovskite.

To visually comprehend the anisotropic and elastic behaviour of the materials under investigation, the Elate Code^40^ has been employed to generate three-dimensional (3-D) contour plots representing Young's modulus (*Y*), linear compressibility (β), shear modulus (*G*), and Poisson's ratio (*ν*). The resulting 3-D visual representations for Cs_2_LiGaBr_6_ and Cs_2_NaGaBr_6_ are depicted in Figs. 3a–d and 4a–d, respectively. It is noteworthy that, except for linear compressibility, all other constants exhibit significant deviations from a completely spherical (3D) morphology, emphasizing the anisotropic nature of these materials.

### Electronic properties

**Figure 5:**
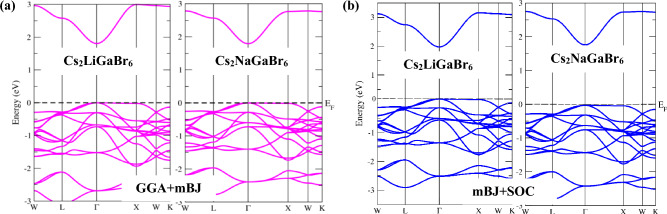
Electronic band strture of Cs_2_MGaBr_6_ calculated through (**a**) GGA + mBJ and (**b**) mBJ + SOC.

The investigation of a material's electronic behavior entails a comprehensive analysis of its band structure, elucidating its electronic properties and discerning whether it demonstrates metallic, semiconducting, or insulating attributes. Henceforth, we conducted an in-depth exploration of the electronic band structure for the materials under scrutiny, employing various exchange–correlation (ε_xc_) functionals, including the generalized gradient approximation (GGA) and the modified Becke-Johnson method (GGA + mBJ) coupled with spin–orbit coupling (SOC). For clarity and convenience, we have presented the band structures calculated using the GGA + mBJ and mBJ + SOC approaches in Fig. 5a,b. Across all these approximations, the materials consistently exhibit semiconductor behavior, characterized by a direct band gap nature (VBM and CBM sharing the same symmetry point Γ). Upon scrutinizing the electronic band structure using these varied approximations, distinct variations in the calculated band gap values are observed, as detailed in Table 3. The GGA approach tends to underestimate the band gap, necessitating the incorporation of the mBJ potential to afford a more precise representation of the band gap^21^. The inclusion of heavier elements mandated the consideration of spin–orbit coupling (SOC) effects. Nonetheless, it is pertinent to note that the introduction of spin–orbit coupling (SOC) alongside the mBJ potential did not elicit significant alterations in the band gap values, with one notable exception observed in the case of Cs_2_LiGaBr_6_, where a modest increase of 0.12 eV has been recorded. Consequently, the GGA + mBJ potential has been strategically chosen for the calculation of all other properties dependent on the band structure throughout this study. The reproduced band gap values with the mBJ approach are found to be 1.82 eV and 1.78 eV for Cs_2_LiGaBr_6_ and Cs_2_NaGaBr_6_, respectively. These values are in reasonably good agreement with analogous studies^16,26^ and are closer to the experimental values of the parent halide perovskite CH_3_NH_3_PbI_3_(Cl, Br)^41^. It is worth noting that these band gap values lie in the specific energy range of 1–1.8 eV, where the release of electrons takes place without generating excessive heat, making these materials effective semiconductors for solar energy conversion. Further analysis of the band structure provides valuable insights into the nature of electronic states, revealing that electronic states in the conduction band (CB) exhibit greater dispersion, while those in the valence band (VB) are less dispersive. This observation suggests that the effective mass of free charge carriers in the VB (holes) is slightly greater than that of carriers in the CB (electrons). This juxtaposition of heavy and light bands proves advantageous in enhancing the thermoelectric performance of Cs_2_MGaBr_6_ halide perovskites. To quantify this, we calculated the effective mass of these carriers by fitting the conduction and valence bands to the parabolic equation, given by^42^: ${m}^{*}={h}^{2}$[($\frac{{\partial }^{2}\varepsilon (\lambda )}{{\partial\uplambda }^{2}}$]^-1^ Where *k* represents the wave vector, and ε($(\lambda$) represents the eigenvalue corresponding to the band edge. The subsequent effective mass values, documented in Table 3 alongside comparable published findings, reveal notable agreement with other comparable results^16,26^. Importantly, the effective mass of carriers for the materials under study is significantly below 0.20. This lower effective mass is advantageous for achieving enhanced carrier mobility, a highly desirable feature for the development of efficient electronic and optoelectronic devices.

**Table 3:** Calculated band gap values, effective mass of the carriers for the present materials in comparison with other published findings.

Parameter	Band gap (eV)			Other works	${m}_{e}^{*}$	${m}_{h}^{*}$	Other works
Approximation	GGA	GGA + mBJ	mBJ + SOC	Theory	mBJ	mBJ	Theory
Cs_2_LiGaBr6	0.74	1.82	1.94	1.96^26^, 1.81^16^	0.163	0.181	0.156^26^, 0.176^16^
Cs_2_NaGaBr6	0.48	1.78	1.74	1.76^26^, 1.82^16^	0.184	0.190	0.176^26^, 0.186^16^

#### Density of states

**Figure 6:**
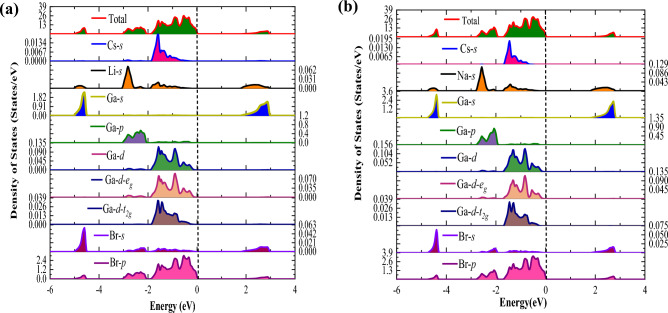
Plot of the electronic density of states for (**a**) Cs_2_LiGaBr_6_ and (**b**) Cs_2_NaGaBr_6_ halide perovskites.

After elucidating the electronic band structure, our focus now shifts to the analysis of the density of states, aiming to understand the involvement of electronic states in shaping the electronic characteristics of these materials. For this purpose, we conducted calculations for the density of states, encompasssing both the total and partial density of states, as skechted in Fig. 6a,b. For Cs_2_MGaBr_6_, the orbital contributions of various valence states, including Cs-6*s*, Li-2*s*, Na-3*s*, Ga-4*s*, 4*p*, 3*d*, 3*d-e*_*g*_, 3*d-t*_*2g*_, and Br-4*s*, Br-4*p* is shown in Fig. 6a,b. Notably, Ga-4* s* states and Br-4*p* states emerge as the most significant contributors in shaping the electronic profile of these materials. From Fig. 6a,b, Ga-4*s* states are positioned in the energy range of -6 to -4 eV within the valence band, while their antibonding states occupy the conduction band within the energy range of 2 to 4 eV. Furthermore, we witness that Br-4*p* states acquire electrons from Cs-6*s*, Na-3*s*, and Ga-4*s*, 4*p* states to complete their orbitals, positioning themselves in the valence band just below the Fermi level in pursuit of exchange energy, leading to the emergence of the band gap.

### Thermodynamic features

**Figure 7:**
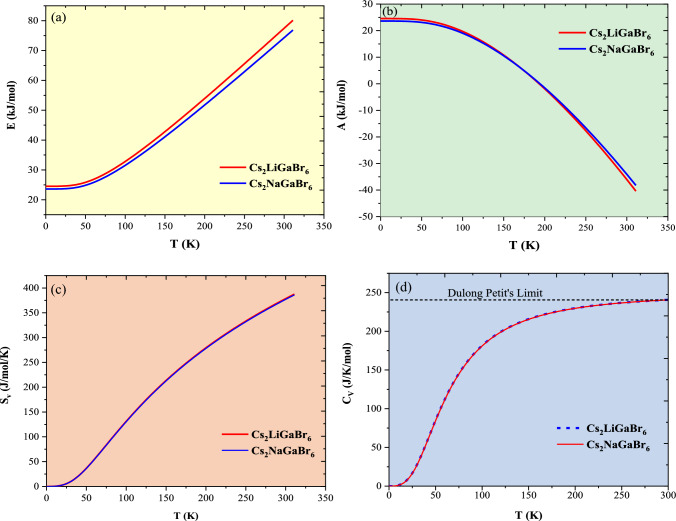
Thermodynamic coefficients of Cs_2_MGaBr_6_ perovskites as a function of temperature (**a**) fluctuations in vibrational internal energy (E in ${\text{kJ}}/{\text{mol}}$), (**b**) the vibrational Helmholtz free energy (A in ${\text{kJ}}/{\text{mol}}$), (**c**) the vibrational entropy (*S*_*V*_ in ${\text{J}}/{\text{K}}/{\text{mol}}$), and (**d**) the constant-volume heat capacity (*C*_*V*_ in ${\text{J}}/{\text{K}}/{\text{mol}}$).

The exploration of thermodynamic functions involving vibrational contributions aims to comprehend properties such as variations in vibrational internal energy (*E*), vibrational Helmholtz free energy (*A*), vibrational entropy (${S}_{V}$), and specific heat at constant volume (C_v_) concerning temperature (T). These thermodynamic properties are predicted using the quasi-harmonic Debye model^43,44^. In accordance with this model, the formulations for vibrational Helmholtz free energy (*A*), vibrational entropy (${S}_{V}$), Debye temperature (${\theta }_{D}$), and specific heat at constant volume (C_v_) are given as follows:

$$\begin{aligned} & {A_{{\text{Vib}}}}\left( {\theta \left( V \right);\;T\; = \;n{k_B}T\;\frac{{9{\theta_D}}}{8T} + 3\ln 1 - {e^{\frac{\theta D}{T}}} - D\left( {\frac{{\theta_D}}{T}} \right.} \right) \\ & {{\uptheta }_{\text{D}}}{\text{\;}} = {\text{\;}}\frac{h}{{K_B}}{\left( {6{\pi^2}{V^\frac{1}{2}}N} \right)^\frac{1}{3}}f\left( v \right)\sqrt {\frac{{B_s}}{M}} \\ & {S_V} = n{k_B}T\;4D\left( {\frac{{\theta_D}}{T}} \right) - 3\ln 1 - {e^{\frac{{\theta_D}}{T}}} \\ & {\text{Cv}} = 3nk\left( {4D\frac{{\theta_D}}{T} - \frac{{\frac{{3{\theta_D}}}{T}}}{{e{\theta^\frac{D}{T}}}}} \right) \\ \end{aligned}$$

where *n* signifies the number of atoms per formula unit, ${k}_{B}$ is the Boltzmann constant, ${B}_{s}$ represents the adiabatic Bulk modulus, $M$ denotes molecular mass per unit cell, and $D$ is the Debye integral. The temperature-dependent fluctuations in vibrational internal energy, Helmholtz free energy, vibrational entropy, and specific heat at constant volume are visually illustrated in Fig. 7a–d. Initially, we explore vibrational internal energy, recognizing the established phenomenon that continuous heat input leads to an escalation in the kinetic energy of constituent atoms, resulting in heightened atomic vibrations and, consequently, an increase in vibrational internal energy. The observed linear increase of *E* with temperature (T), as depicted in Fig. 7a, signifies a proportional rise in the system's enthalpy. The variation in vibrational Helmholtz free energy (*A*), as depicted in Fig. 7b, manifests a decrease in *A* with increasing temperature (*T*) for both materials. This observed trend substantiates that the thermal energy available for useful work remains nearly constant for both materials. The inherent disorder within the system, quantified as entropy (${S}_{V}$), escalates down the periodic table group (from Li to Na) due to an expansion in atomic size, allowing for a greater potential orientation of subatomic particles. Figure 7c illustrates that ${S}_{V}$ is nearly identical for both materials owing to minor differences in their atomic sizes, and it rises with temperature (T), indicating an augmentation in thermally accessible vibrational states.

In the context of thermodynamics and statistical studies^45,46^, heat capacity (C_V_) is crucial for understanding lattice vibrational characteristics. The C_V_ plot behaviour, depicted in Fig. 9d, is divided into two regions. At temperatures significantly lower than the Debye temperature (${\theta }_{D}$), it adheres to the rule: ${C}_{V}=\frac{12}{5}{\pi }^{4}nR-{\left(\frac{T}{{\theta }_{D}}\right)}^{3}$. Conversely, at elevated temperatures (T >  > ${\theta }_{D}$), the curve conforms to the expression: ${C}_{V}\cong 3nR$, following Dulong–Petit’s limit^47^. Here, *n* represents the number of atoms per formula unit, R is the universal gas constant, and ${\theta }_{D}$ is the Debye temperature at 0 K. The determined values for the Debye Temperature (${\theta }_{D}$) (Kelvin), Zero-point energy (*E*_0_), expressed in kilojoules per mole, and the Gruneisen parameter γ (dimensionless) are provided in Table 4.

**Table 4:** Computed values for the Debye Temperature (${\theta }_{D}$) measured in Kelvin, Zero Point Energy (*E*_*0*_) expressed in kilojoules per mole, and the Gruneisen Parameter (γ) (dimensionless).

Parameter	${\theta }_{D}$	*E*_*0*_	γ
Cs_2_LiGaBr_6_	263	24	2.23
Cs_2_NaGaBr_6_	252	23	2.17

The analysis of the specific heat (C_V_) plot depicted in Fig. 7d confirms the adherence of Cs_2_MGaBr_6_ compounds to the above-mentioned rules. Notably, at temperatures (*T*) considerably below the Debye temperature (${\theta }_{D}$), the observed increase in ${C}_{V}$ aligns proportionally with ${T}^{3}$, affirming conformity with Debye's low-temperature specific heat law, commonly referred to as Debye's ${T}^{3}$ law. Conversely, at temperatures significantly surpassing ${\theta }_{D}$, ${{\text{C}}}_{{\text{V}}}$ remains nearly constant for both these systems, approaching the Dulong-Petit’s limit. This phenomenon signifies that, at elevated temperatures, our computational results are in accordance with the classical thermodynamics encapsulated by the Dulong–Petit’s law. The tabulated values presented in Table 5 unveil a discernible decreasing trend following the sequence Cs_2_LiGaBr_6_ > Cs_2_NaGaBr_6_. This trend is ascribed to compounds with heavier atoms exhibiting a diminished velocity of sound (c) attributable to an increase in density. Additionally, the dimensionless Gruneisen parameter (γ), calculated as γ = $\frac{3(1+ \epsilon )}{2(2- \epsilon )}$, serves as an insightful metric predicting the anharmonic properties of the solid material. Remarkably, this parameter demonstrates noteworthy uniformity for both the compounds, implying a consistent trend in thermodynamic properties for the scrutinized perovskites (Cs_2_MGaBr_6_). This uniformity suggests that these compounds are anticipated to exhibit cohesive thermodynamic behaviour.

**Table 5:** Calculated polaronic parameters in Cs_2_MGaBr_6_ halide perovskites.

Configuration	${\omega }_{LO}({{\text{cm}}}^{-1})$	${\varepsilon }_{Static}$	${\varepsilon }_{\infty }$	α	${r}_{p}$ (Å)	${m}_{p}({m}_{0})$
Cs_2_LiGaBr_6_	122.32	5.79	2.64	4.73	76.43	0.92
Cs_2_NaGaBr_6_	145.58	5.94	2.81	3.42	67.25	0.85

### Thermoelectric coefficients

**Figure 8:**
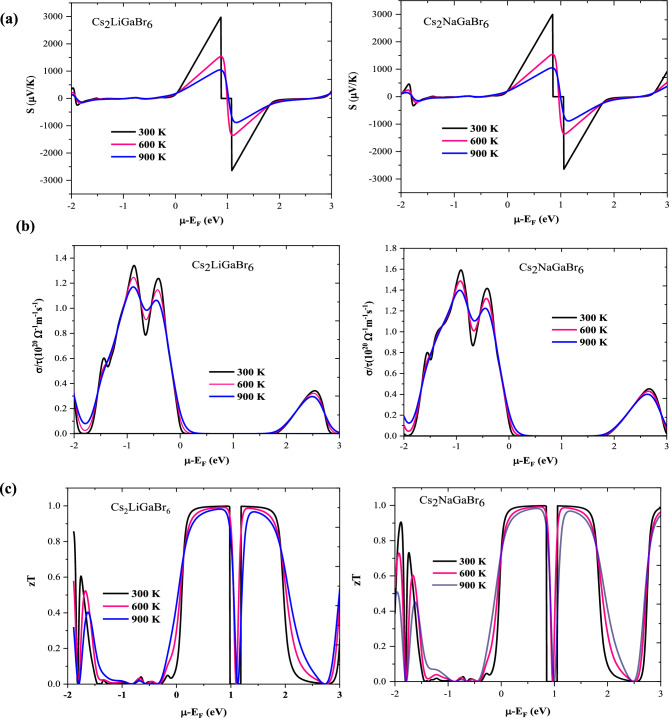
Plot of thermoelectric coefficients (**a**) Seebeck coefficient (S), (**b**) electrical conductivity (σ/τ), (**c**) figure of merit (zT) as a function of chemical potential and temperature for Cs_2_MGaBr_6_ halide perovskites.

**Figure 9:**
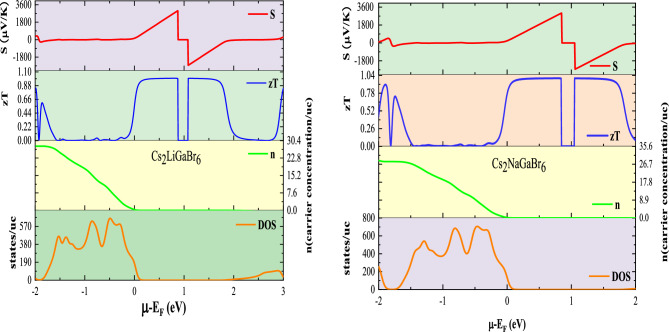
Comparative variation in Seebeck coefficient (S), figure of merit (zT), and carrier concentration (n), alongside volumetric density of states (DOS) at a temperature of 300 K, relative to the chemical potential for Cs_2_MGaBr_6_ halide perovskites.

In this section, the thermoelectric coefficients of the given materials have been analysed to assess their potential for waste heat recovery applications. These coefficients, which encompass the Seebeck coefficient (S), electrical conductivity (σ), and the figure of merit (zT), have been derived through the utilization of the BoltzTraP code^48^. We investigated the variations of these coefficients against chemical potential (− 2 < $\upmu$  > 3) at different temperatures (300 K, 600 K, 900 K), as illustrated in Fig. 8a–c. Seebeck coefficient (S) elucidates the magnitude of thermoelectric voltage generated in response to a temperature gradient across the material. We have computed the Seebeck coefficient for the given materials in response to chemical potential at different temperatures, as illustrated in Fig. 8a. In semiconductor materials, the alignment of the chemical potential with the Fermi level is governed by the Fermi–Dirac distribution function, wherein the distinction between positive and negative values delineates the *n*-type (conduction band) and *p-*type (valence band) regions, respectively. For the given materials, we observe a positive value of the Seeback coefficient (S) at $\upmu$=0, indicating their *p*-type nature, consistent with the electronic band structure analysis showing a higher density of states in the valence band than in the conduction band. As illustrated in Fig. 8a, at room temperature, peak values of the Seebeck coefficient are observed within the *n*-type doping region, measuring 3000 $\mu V {{\text{K}}}^{-1}$ for Cs_2_LiGaBr_6_ and 2850 $\mathrm{\mu V }{{\text{K}}}^{-1}$ for Cs_2_NaGaBr_6_, respectively. These values are impressively superior to state-of-the-art materials due to the favourable band gap and the increased effective mass of hole carriers, which predominantly enhance the Seebeck coefficient of these halide perovskites^17,24,25^. At elevated temperatures, the Seebeck values decrease to 1000 $\mathrm{\mu V }{{\text{K}}}^{-1}$ for Cs_2_LiGaBr_6_ and 850 $\mathrm{\mu V }{{\text{K}}}^{-1}$ for Cs_2_NaGaBr_6_, respectively. The differences in the Seebeck coefficient or thermopower among these materials can be expounded upon by taking into account the band gap, which is susceptible to alterations due to changes in both temperature and carrier concentration. It is crucial to emphasize that thermopower exhibits a remarkable sensitivity to the unique attributes of the density of states (DOS) in proximity to the band edge. Notably, any elevation in temperature directly influences the density of states, thereby exerting a consequential effect on the thermopower characteristics. To explore the correlation between electronic and transport properties, we computed graphical plots illustrating the Seebeck coefficient, carrier concentration (*n*), and volumetric density of states (DOS) at 300 K for both materials. The comparative behaviour of these parameters against chemical potential in proximity to the Fermi level is depicted in Fig. 9. The pronounced peaks in the Seebeck coefficient observed within the energy range of 0.5 to 1.0 eV near the Fermi level are ascribed to the diminishing density of states and optimal carrier concentration, thereby maintaining a favourable temperature gradient. In contrast, a departure from the Fermi level results in increased peaks in the DOS, leading to a reduction in the Seebeck coefficient.

As depicted in Fig. 8a, the Seebeck coefficient (S) exhibits a notable decline against temperature variations since thermal energy of carriers increases which increases carrier concentration. This observed variation in Seebeck coefficient, characterized by both increments and decrements in response to changes in carrier concentration, serves as a distinctive hallmark of bipolar conduction. In this context, it is worth noting that both hole and electron carriers substantially contribute to the charge transport, consistently demonstrated across different fixed temperature conditions, as visually depicted in Fig. 7a. This phenomenon can be aptly described by the relationship ${\text{S}}\propto (\frac{1}{{\text{n}}}{)}^\frac{2}{3}[$^21^$]$. Nonetheless, it appears that bipolar conduction exerts a limited influence on the behaviour of the present materials, as their band gap values exceed the critical threshold of 0.64 eV typically associated with the occurrence of bipolar conduction.

Figure 8b presents the electrical conductivity per relaxation time (σ/τ) plotted as a function of the chemical potential (μ), offering insights into the material's electrical conductance originating from electron transport in response to temperature gradients, moving from regions of higher to lower temperatures. Notably, while examining the peak values of (σ/τ) concerning the chemical potential, we observe relatively consistent behaviour across different temperature settings for both compounds. There are distinct dual curves, each featuring two peak values of σ/τ within their respective *p*-type and *n*-type regions. At 300 K, the peak values of $1.38\times$ 10^20^ (${\Omega }^{-1}{{\text{m}}}^{-1}{{\text{s}}}^{-1}$) and $1.55\times$ 10^20^ (${\Omega }^{-1}{{\text{m}}}^{-1}{{\text{s}}}^{-1}$) are obtained for the* p*-type region. The attainment of decent electrical conductivity values can be attributed to the lower effective mass of electrons within the conduction band (CB) region. This relationship stems from the fact that electrical conductivity (σ) is directly proportional to the carrier concentration (n), expressed as: $\upsigma =\mathrm{ ne\mu }$, and inversely proportional to (${{\text{m}}}^{*}$)^(5/2)^ with (${{\text{m}}}^{*}$) denoting the effective mass of the carriers.

Therefore, temperature variations positively influence electrical conductivity by increasing the thermal energy of carriers, consequently augmenting carrier concentration. Furthermore, it's noteworthy that electrical conductivity consistently ceases at $\upmu$=0 within the energy range of (0–1.8) eV, which ensues due to the absence of electronic states in this specific range, as graphically illustrated in Fig. 9. Consequently, there is a deficit of charge carriers, leading to a complete cessation of electrical conductivity in this specific energy range.

**Figure 10:**
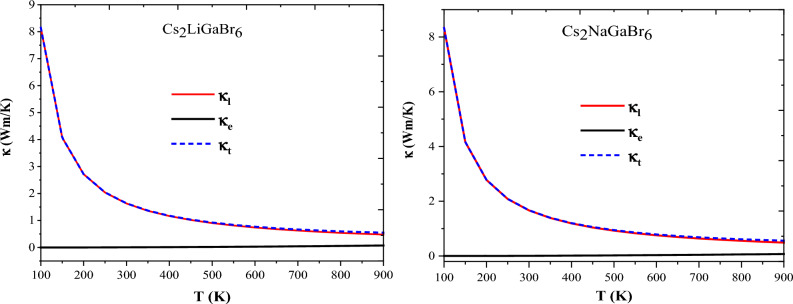
Variations in lattice (κ_l_), electronic (κ_e_) and total thermal conductivity (κ_t_) against selected range of temperature for Cs_2_MGaBr_6_ halide perovskites. [Lattice thermal conductivity (κ_l_) has been computed using the Gibbs2 code, while electronic thermal conductivity (κ_e_) was calculated via the BoltzTraP code].

The thermal conductivity (κ_t_) of a material comprises contributions from both electronic and lattice components, expressed as: κ_t_ = κ_e_ + κ_l_. Electronic thermal conductivity (κ_e_) arises from the thermal energy carried by electrons, while lattice thermal conductivity (κ_l_) results from the lattice vibrations. In the present work, we aimed to calculate both lattice and electronic thermal conductivity, alongside determining the overall thermal conductivity. The calculation of electronic thermal conductivity has been performed using the BoltzTraP code^48^, while the lattice thermal conductivity has been determined using Slack's model. Slack's model, expressed as:^21,49^ ${\upkappa }_{{\text{l}}}=\frac{{\text{A}}{\uptheta }_{{\text{D}}}^{3}{{\text{V}}}^{1/3}{\text{m}}}{{\upgamma }^{2}{{\text{N}}}^{2/3}{\text{T}}},$ underscores that the lattice thermal conductivity (κ_l_) is influenced by various factors, including the Debye temperature (${\uptheta }_{{\text{D}}}$), Gruneisen parameter ($\upgamma$), temperature (*T*), volume (*V*), average molar mass per atom (*m*), and the number of atoms per unit cell (*N*). The parameter A is determined as^49^: ${\text{A}}=\frac{2.43\times {10}^{8}}{1-\frac{0.514}{\upgamma }+\frac{0.228}{{\upgamma }^{2}}}$. Employing these pertinent variables, the Slack model has been employed to compute the lattice thermal conductivity of the present materials. The graphical representation of lattice ${(\upkappa }_{{\text{l}}})$, electronic (κ_e_) and total thermal conductivity (κ_t_) for the given materials is presented in Fig. 10. In this context, we observe a decrease in lattice thermal conductivity with rising temperature, attributed to the increased anharmonic phonon scattering due to the clustering of heavier elements, a phenomenon known as the 'rattling effect'. Conversely, electronic thermal conductivity exhibits an upward trend with increasing temperature because higher temperatures augment the thermal energy of carriers, thereby enhancing electronic thermal conductivity. At lower temperatures, such as 300 K, the lattice component is observed to predominate over the electronic contribution. However, as the temperature increases, reaching approximately 900 K, both the lattice thermal conductivity and the overall conductivity exhibit a uniform decrease. The calculated values for lattice thermal conductivity are 2.0 ${\text{W}}/{\text{mK}}$ and 1.5 ${\text{W}}/{\text{mK}}$ at 300 K, which are notably low, indicating the potential for improved thermoelectric performance of these materials. Furthermore, the lower values of κ_e_ at room temperature further imply that the specified materials may exhibit favourable thermoelectric performance.

The figure of merit (zT) is a crucial metric for evaluating the thermoelectric performance of materials, with a value approximately equal to or greater than one indicating promising potential for thermoelectric device applications. Figure 8c illustrates the dependence of the thermoelectric figure of merit (zT) on chemical potential at various temperatures for the specified compounds. Peak values of zT, reaching 1.08 for Cs_2_LiGaBr_6_ and 1.04 for Cs_2_NaGaBr_6_, are observed within the energy range of 0.5–1.0 eV, likely attributed to significant peaks in the Seebeck coefficient within this region. Considering that the figure of merit (zT) is the square of the Seebeck coefficient, it inherently reflects peaks corresponding to those observed in the Seebeck coefficient, as depicted in Fig. 9. The notably high values of zT endorse these materials for applications in renewable energy and thermoelectric devices.

### Optical properties

**Figure 11:**
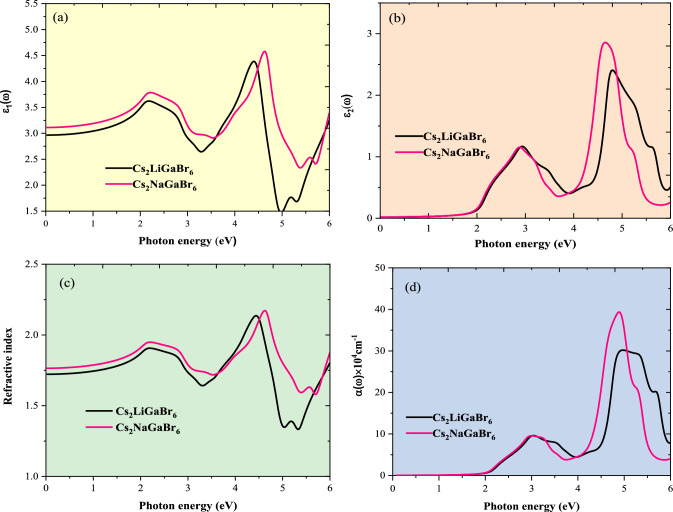
Plot of optical parameters against photon energy for Cs_2_MGaBr_6_ halide perovskites: (**a**) real part of dielectric, (**b**) Imaginary part of dielectric, (**c**) refractive index, and (**d**) absorption coefficient.

The presence of direct band gaps in these materials, spanning the visible and infrared spectra, motivates a comprehensive exploration of their optical properties. Consequently, in this section, we present key optical properties, including the dielectric function, refractive index and absorption spectra^50^. The dielectric function, denoted as ε(ω), elucidates a material's response to electromagnetic radiation, comprising two components ε_1_(ω) and ε_2_(ω), representing the real and imaginary facets respectively, which vary with frequency^50^. In Fig. 11a,b, we present the computed spectra of ε_1_(ω) and ε_2_(ω) for the given compounds. In the energy range of 0 to 6 electron volts (eV), both spectra display similar patterns, attributed to the involvement of Br-*p* and Ga-*s* states in the valence and conduction bands, respectively. The static dielectric constants (ε_0_) for Cs_2_LiGaBr_6_ and Cs_2_NaGaBr_6_ are determined to be 4.3 and 4.55, respectively. A higher ε_0_ value suggests a reduced probability of charge carrier recombination, signifying enhanced performance in optoelectronic applications. The imaginary part, ε_2_(ω), depicted in Fig. 11b, demonstrates absorptive behaviour and provides insights into the band structure. The threshold values for Cs_2_LiGaBr_6_ and Cs_2_NaGaBr_6_ closely align with the electronic bandgap values calculated via the TB-mBJ method. Another crucial optical property that offers insights into light-material interactions is the refractive index. As light traverses’ diverse media, its velocity undergoes alterations, resulting in variations in the refractive index of the material. From Fig. 11c, the static refractive index values are measured as 2.1 for Cs_2_LiGaBr_6_ and 2.2 for Cs_2_NaGaBr_6_, respectively, indicating excellent optical performance for these materials. Absorption spectra offer valuable insights into a material's potential for efficient solar energy conversion. In Fig. 11d, we present the calculated absorption spectra, denoted as α(ω), spanning from 0 to 6 electron volts (eV), for Cs_2_LiGaBr_6_, and Cs_2_NaGaBr_6_. These spectra exhibit two distinct absorption peaks for each compound, characterized by an increasing trend attributed to electronic transitions from bonding states to anti-bonding states. The initial peak, located within the visible energy range at approximately 3.0 eV for both compounds, is followed by a notable deviation or "kink" occurring around 3.5 eV. The prominent peak, located at approximately 5 eV, demonstrates absorption coefficients α(ω) of ($$30\times 10$$^4^ cm^−1)^ and ($$40\times 10$$^4^$$)$$ cm^−1^ for Cs_2_LiGaBr_6_ and Cs_2_NaGaBr_6_, respectively. These values, notably high and falling within the visible spectrum, suggest the potential of these materials as effective alternatives to lead (Pb) halide perovskites, especially in photovoltaic (PV) applications.

### Phonon stability and electron–phonon coupling strength

**Figure 12:**
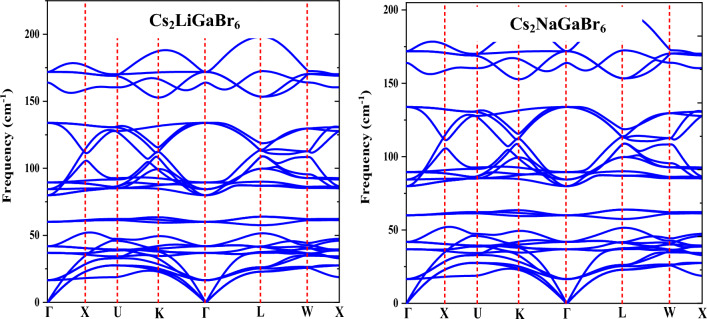
Phonon band structures of Cs_2_MGaBr_6_ (M = Li, Na) halide double perovskites.

Evaluating the dynamical stability of a material holds critical importance in assessing its viability for high-performance device applications. To achieve this, phonon band structure calculations have been computed at operational temperatures using density functional perturbation theory (DFPT) within the pseudopotential-based Quantum espresso package^51^. The resulting phonon band structures for the specified materials are presented in Fig. 12. For Cs_2_MGaBr_6_, each unit lattice comprises 10 atoms, resulting in a total of 30 vibration modes. These modes include 3 acoustic branches and 27 optical branches, collectively exhibiting symmetry at the center of the Brillouin zone, represented as: Γ = 10${E}_{u}$ +${5A}_{2u}$ + ${9T}_{g}$ + ${3T}_{u}$ + ${2E}_{g}$+ ${A}_{1g}$, with 5${E}_{u}$+ ${6T}_{g}$ + ${2T}_{u}$ +${E}_{g}$ being degenerate. Notably, among these vibration modes, 8${E}_{u}$ + 4${A}_{2u}$ + ${3T}_{u}$ are active in the infrared region, while ${9T}_{g}$ + ${2E}_{g}$ + ${A}_{1g}$ modes are Raman active. Three acoustic modes, 2${E}_{u}$ + ${A}_{2u}$, exhibit no activity due to the absence of quadratic functions. Upon examination of the phonon bands (Fig. 12), the LO (Longitudinal Optical) phonon frequencies are observed to span three distinct ranges: 20–60 cm^−1^, 80–140 cm^−1^, and 150–190 cm^−1^. In the low-frequency range (20–60 cm^−1^), significant contributions come from Cs atoms, primarily attributed to the wagging motion between Cs and Br atoms. In the intermediate frequency range (80–140 cm^−1^), interactions between Br atoms and two types of metal atoms predominate the phonon states. Lastly, the high-frequency range (150–190 cm^−1^) is characterized by stretching vibrations between Ga and Br atoms, resulting in fewer phonon states.

The impact of electron–phonon coupling on a material's physical and chemical properties remains an intriguing paradox. It has been observed that in the case of polar semiconductors, such as halide perovskites, the interaction between charge carriers and the macroscopic electric field generated by longitudinal optical (LO) phonons, known as the Frohlich interaction, plays a central role in scattering mechanisms, especially near room temperature^52^. In this study, we utilized the Frolich polaron model to investigate the electron–phonon coupling in Cs_2_MGaBr_6_ compounds. The dimensionless Fröhlich parameter α, representing dielectric electron–phonon coupling and serving as a comparative measure of Fröhlich coupling strength, is expressed as^52^:

$$\mathrm{\alpha }=\frac{1}{4\pi \theta {\varepsilon }_{0}}\frac{1}{2}\left(\frac{1}{{\varepsilon }_{\infty }}-\frac{1}{{\varepsilon }_{static}}\right)\frac{{e}^{2}}{h{\omega }_{LO}}{\left(\frac{2{m}^{*}{\omega }_{LO}}{h}\right)}^{1/2}$$

Here, the symbol ${\omega }_{LO}$ signifies the characteristic phonon angular frequency specific to the respective materials, while the term $\left(\frac{1}{{\varepsilon }_{\infty }}-\frac{1}{{\varepsilon }_{static}}\right)$ effectively quantifies the strength of the interaction between carriers and the lattice in ionic crystals. In the context of our double halide perovskites, there exist numerous phonon branches that interact with electrons. To account for this complexity, we computed an effective longitudinal optical phonon frequency by taking a spectral average over all the infrared active optical phonon branches^53^. The mean frequency of Longitudinal Optical (LO) modes is determined by utilizing the infrared (IR) phonon frequencies obtained through density functional perturbation theory (DFPT). In this context, the interaction between free electrons and the lattice involves multiple infrared-active optical phonon modes. To obtain an effective longitudinal optical phonon frequency (ω_LO_), we apply the Hellwarth and Biaggio ansatz^53^. The oscillator strength (*W*_i_) of the LO phonon modes is computed using the LO-TO (Transverse Optical) splitting, as described by the following equation: ${W}_{e}^{2}=\frac{1}{{\varepsilon }_{\infty }}({\omega }_{LO,i}^{2}-{\omega }_{TO,i}^{2}$) The process involves calculating the quadratic mean of the single oscillator strengths (${W}^{2}=\sum {W}_{i}^{2}$) to determine the oscillator strength of the individual phonon branch, denoted as *W*. Subsequently, the frequency ω_LO_ (measured in wave numbers) for this branch is computed by solving the equation:

$$\frac{{W}^{2}}{{\omega }_{LO}}cot h\left(\frac{hc{\omega }_{LO}}{{2}_{KB}T}\right)=\sum_{i=1}^{2}\frac{ {W}_{i}^{2}}{{\omega }_{LO,i}}cot h\left(\frac{hc{\omega }_{LO},i}{{2}_{KB}T}\right)$$

The computed values of mean frequency of Longitudinal optical modes (LO) and electron–phonon coupling parameter ($\mathrm{\alpha })$ are shown in Table 5. To further illustrate the polaron characteristics of these double halide perovskites, we have calculated the corresponding polaron radius and effective mass of polarons using the Feynman polaron theory^54^:

$${r}_{p}=3/0.44\alpha {)}^\frac{1}{2}(2{m}_{e}{\omega }_{LO}{)}^{-1/2}$$

$${m}_{p}=\frac{1}{6}\alpha +\frac{1}{40}{\alpha }^{2}$$

The computed results presented in Table 5 illustrate significant Fröhlich coupling constants ranging from 3 to 5 for these double halide perovskites. These values surpass typical values observed in conventional halide perovskites, which generally hover around 2^55^. This observation suggests a stronger electron–phonon coupling effect in these double halide perovskite structures, characterized by alternating arrangements of different metal-halogen octahedra.

### Theoretical models

First-principles calculations, utilizing an all-electron full-potential model within the density functional theory (DFT) framework, were exclusively performed with the Wien2k simulation package^56^, to investigate the structural, electronic, optical, and thermoelectric properties of Cs_2_MGaBr_6_ halide perovskites. To begin, we constructed the structural model of the materials and utilized the Perdew, Burke, and Ernzerhof generalized-gradient approximation (PBE-GGA) for geometry optimization^57^. Subsequently, we refined our calculations by integrating sophisticated techniques, particularly utilizing the modified Becke Johnson (mBJ) semi-local exchange potential in conjunction with spin-orbit coupling to precisely ascertain the electronic structure and explore the thermoelectric and optical properties of the specified material^58^. In the full-potential model, the crystal unit cell is divided into two main regions: the "muffin-tin region," where the wave function is expanded using spherical harmonics, and the "interstitial region," where the wave function is expanded as plane waves. A cutoff energy of − 6.0 Ry was applied to differentiate core states from valence states in our computations. To attain self-consistency, a grid of 1300 *k*-points within the first Brillouin zone (BZ) was employed, alongside parameter specifications including R_MT_K_max_ = 7, a wave function expansion up to *l*_max_ = 10, and a charge density expansion of G_max_ = 12 atomic units per angstrom, were consistently implemented. We deemed our calculations to be converged upon the attainment of atomic forces at 10^–5^ Ry/Bohr and a charge reaching a value 0.0001 |e|. The thermoelectric coefficients have been determined through the application of Boltzmann transport theory, coupled with the rigid-band model and the constant-scattering time approximation (CSTA), as implemented in the BoltzTraP code^43^. The Seebeck coefficient (S), electrical conductivity (σ), and elctronic thermal conductivity (*k*_e_) have been calculated as a function of the chemical potential (µ) for the specified perovskite materials, employing established formulations as outlined below^48^:

$$\begin{aligned} & S = \frac{{e{k_B}}}{\sigma }\int {\left( { - \frac{{\partial {f_0}}}{\partial \varepsilon }} \right)\frac{\varepsilon - \mu }{{{k_B}T}}} \Xi (\varepsilon )d\varepsilon \\ & \sigma = {e^2}\int {\left( { - \frac{{\partial {f_0}}}{\partial \varepsilon }} \right)} \Xi (\varepsilon )d\varepsilon \\ & {k_e} = k_B^2T{\int {\left( { - \frac{{\partial {f_0}}}{\partial \varepsilon }} \right)\left( {\frac{\varepsilon - \mu }{{{k_B}T}}} \right)}^2}\Xi (\varepsilon )d\varepsilon \\ \end{aligned}$$

Here, $\Xi (\varepsilon )$ is the transport distribution function, denoted by $\Xi^{{\alpha ,\beta }}(\varepsilon ) = \sum\limits_k {\delta (\varepsilon - {\varepsilon_k})v_k^\alpha } v_k^\beta {\tau_k}$ where $v_k^\alpha$ represent *α*th component of the group velocity with wave vector *k*. To enhance accuracy, the *k-*mesh was expanded to include 100,000 *k*-points. The optical properties of the given materials have been evaluated using the random phase approximation (RPA) method^59^. Within this framework, various optical parameters, including the joint density, Dirac delta function, and complex dielectric function, are computed and solved^59^. Subsequent parameters have been derived using a dense mesh of 5000 *k*-points. The complex dielectric function*,* denoted as ε(ω), is defined as the sum of its real part, ${\varepsilon }_{1}\left(\omega \right)$, and the imaginary part, $i{\varepsilon }_{2}\left(\omega \right)$. The Kramers–Kronig relation interconnects the real and imaginary parts of the dielectric function^50^. The imaginary part $i{\varepsilon }_{2}\left(\omega \right)$ can be determined by using this equation: ${\varepsilon }_{2}\left(\omega \right)= \frac{8}{2\mu {\omega }^{2}}\sum nn{\prime}{\int }_{BZ}^{0}{\left|Pnn{\prime}(k)\right|}^{2}\frac{d{s}_{k}}{\nabla {\omega }_{n{n}{\prime}}(k)}$ Here $\left|Pnn{\prime}(k)\right|$ denotes the electric dipole matrix element between n and n' states, ${s}_{k}$ represents the constant value of surface energy, and ${\omega }_{n{n}{\prime}}(k)$ is the energy difference between the two states. The absorption coefficient $(\alpha \left(\omega \right)$ and refractive index ($n\left(\omega \right)$) are determined using certain equations given as^50^:

$$\alpha \left(\omega \right)=\frac{4\pi k(\omega )}{\lambda },n\left(\omega \right)=\frac{1}{\sqrt{2}}{\left[\{{\varepsilon }_{1}(\omega {)}^{2}+{\varepsilon }_{2}(\omega {)}^{2}{\}}^\frac{1}{2}+{\varepsilon }_{1}(\omega )\right]}^{1/2}$$

The computation of phonon spectra across diverse wave vectors has been successfully accomplished through the application of density functional perturbation (DFPT) within the Quantum Espresso Code^51^. This approach involves solving the dynamical matrix to determine phonon frequencies, displacement patterns, dielectric tensors, and effective charges.

The systematic selection of parameters, such as the exchange–correlation functional (PBEsol), Brillouin zone sampling ($5\times 5\times 5$ *k*-points), plane wave cutoff energy (7 *Ry*), and tailored pseudopotentials for individual atomic species, has been meticulously executed to ensure accuracy and consistency in the calculations.

## Conclusion

In summary, we performed first principles calculations of lead-free halide Cs_2_MGaBr_6_ (M = Li, Ga) double perovskites to investigate their necessary stability concerns and possible applications in thermoelectric and optoelectronic domains. These materials manifest the Fm-3 m structural phase stability, as confirmed through tolerance factor assessments and structural optimization simulations. Mechanical stability is assured by satisfying the Born-Huang stability criteria, while both Pugh's ratio and Cauchy's pressure coefficients endorse the brittleness of these perovskites. The analysis of the electronic profile indicates that these compounds exhibit a direct bandgap at the Γ symmetry points, with values of 1.78 eV for Cs_2_LiGaBr_6_ and 1.82 eV for Cs_2_NaGaBr_6_, rendering them well-suited for applications in energy harvesting. Furthermore, the negative enthalpy of formation and precisely defined phonon band structures provide additional evidence of their inherent stability. Employing the Frolich polaron model, we scrutinized electron–phonon coupling interactions, unveiling a robust coupling strength characterized by notable Fröhlich constants falling in the range of 3 to 5. From an application standpoint, these materials exhibit substantial figure of merit (zT), registering values of 1.08 for Cs_2_LiGaBr_6_ and 1.04 for Cs_2_NaGaBr_6_, positioning them as promising candidates for applications in renewable energy and thermoelectric devices. Furthermore, the calculated optical coefficients, encompassing parameters like the dielectric constant, refractive index, and absorption coefficient, manifest peak values within the visible region. Specifically, the elevated absorption coefficient values of $30\times 10$^4^ cm^−1^ for Cs_2_LiGaBr_6_ and $40\times 10$^4^ cm^−1^ for Cs_2_NaGaBr_6_ are observed across visible and infrared spectra, emphasizing their promising applicability in optoelectronic and solar cell technologies.

## Data Availability

The data sets generated and thereafter analysed would be available from the corresponding author upon reasonable request.
